# A study of spatiotemporal delay in hand, foot and mouth disease in response to weather variations based on SVD: a case study in Shandong Province, China

**DOI:** 10.1186/s12889-015-1446-6

**Published:** 2015-01-31

**Authors:** Yilan Liao, Renbin Ouyang, Jinfeng Wang, Bing Xu

**Affiliations:** The State Key Laboratory of Resources and Environmental Information System, Institute of Geographic Sciences and Natural Resources Research, Chinese Academy of Sciences, Beijing, 100101 China; The Key Laboratory of Surveillance and Early-Warning on Infectious Disease, Chinese Center for Disease Control and Prevention, Beijing, 102206 China; Central South University, Changsha, 410083 China; Chang’an University, Xi’an, 710054 China; Jiangsu Center for Collaborative Innovation in Geographical Information Resource Development and Application, Nanjing, 210023 China

**Keywords:** Spatiotemporal delay, Hand, foot and mouth disease, Weather variation

## Abstract

**Background:**

A large number of hand, foot and mouth disease (HFMD) outbreaks was reported during 2008 in China. However, little is known about the effects of meteorological conditions on different temporal and spatial scales on HFMD incidence in children. The aim of this study was to explore the relationship between meteorological data on various temporal and spatial scales and HFMD incidence among children in Shandong Province, China.

**Methods:**

The association between weekly HFMD cases and meteorological data on different temporal and spatial scales in Shandong Province from May 2008 to July 2008 and September 2008 to October 2008 was analyzed, using buffer analysis and the singular value decomposition method.

**Results:**

Wind speed within a 50-km buffer circle of counties in Shandong Province with two-week lag and RH within a 10-km buffer circle of counties with eight-week lag were significantly associated with HFMD incidence. We found a positive correlation between wind speed within the 50-km buffer circle in the prior two weeks and wind speed within the province in the prior one week.

**Conclusions:**

This study revealed strong associations between HFMD incidence in children and wind speed and RH. Thus, meteorological anomalies in the prior two or eight weeks could be used as a valid tool for detecting anomalies during the peak periods of infectious disease.

## Background

Hand, foot and mouth disease (HFMD) is a common infectious disease in children and is caused by a variety of human enteroviruses, such as enterovirus 71 (EV71) and coxsackievirus A16 (Cox A16) [1]. Clinical symptoms of HFMD include fever, blisters and sores in the mouth and on the palms and soles. A minority of patients develop aseptic meningitis, encephalitis, acute flaccid paralysis, neurogenic pulmonary edema, and myocarditis. Severe disease in individuals with rapid progression can lead to death [2]. In 1957, the first HFMD case was discovered in New Zealand [3]. Global HFMD prevalence rates have increased from 125.5 in 2001 to 435.9 in 2007 [4]. China is one of the countries with the highest incidence of HFMD [5]. HFMD has been upgraded to a Class C communicable disease since 2008 in China. There were 83,344 HFMD cases in 2007, and 17 patients died of the disease. Approximately 40,000 patients were from Shandong Province, constituting half of the country’s total cases [6]. There is no effective vaccine against HFMD, so people have no choice but to adopt corresponding prevention and control measures [7]. Therefore, exploring the correlations between HFMD incidence and meteorological elements is of great significance in improving early warnings and control of the disease.

Currently, there is national and international research being undertaken regarding the influence of meteorological factors on HFMD incidence. Ma investigated the HFMD visiting rate and meteorological factors during 2000–2009 in Hong Kong, reporting that the visiting rate was influenced by average wind speed and relative humidity (RH) over the prior two weeks [8]. Onozuka studied average temperatures and RH effects on HFMD during 2000–2010 in Japan and found that when these factors increased, so did the prevalence rate of HFMD [9]. Zhang analyzed monthly meteorological factors and HFMD data in the city of Kaifeng from 2008 to 2010 and noted correlations between HFMD incidence and average temperature, RH and precipitation [10]. There have been many mathematical models used in these studies to describe the relationships between HFMD incidence and meteorological factors. Wang used a susceptible restore transmission model [11], Hii used a Poisson regression model [7], and Hu used a geographically weighted regression model [12]. Most scholars have established databases using statistical software to perform correlation and regression analyses [8,13]. Considering the various influences on infectious disease using different temporal and spatial scales [14], such as the viral incubation period and the potential delay in parental awareness of clinical disease symptoms in children [15], factors that affect disease have certain time lags. However, few scholars have researched these questions. This study used buffer analysis and the singular value decomposition (SVD) method to explore the relationships between HFMD incidence and meteorological factor anomalies (average wind speed, average RH, precipitation, average temperature and temperature difference) on various temporal and spatial scales in Shandong Province. SVD is a common meteorological research method. Because of its solid mathematical foundation and simple principles and calculation and the easily comprehensible physical meaning of the coupling signal, we used SVD to explore the relationships between HFMD incidence and meteorological factors. We found that the HFMD incidence in Shandong Province was positively correlated with wind speed in the prior two weeks within a 50-km buffer circle, and it was negatively correlated with RH in the prior eight weeks within a 10-km buffer circle. There were positive correlations with wind speed during the prior two weeks within the 50-km buffer circle in the province and with wind speed in the counties of Shandong during the prior one week. This paper thereby provides a reference for the short-term prediction of HFMD incidence in Shandong Province.

## Methods

### Study site

Shandong Province lies between 34.25° and 38.23°N and between 114.36° and 122.43°E on China’s eastern coast, with a population density of 589 per km^2^. The province is located on the eastern edge of the North China Plain and in the lower reaches of the Yellow River (Huang He), and it extends out to the sea in the form of the Shandong Peninsula. The northwestern, western, and southwestern parts of the province are all part of the vast North China Plain. The center of the province is more mountainous. The eastern part of the province is the hilly Shandong Peninsula, which extends into the sea. Shandong has a warm, temperate monsoon climate. The climatic characteristics in the province are that it is dry and windy in the spring, hot and rainy in the summer, cool in the autumn, and dry and cold in the winter. The provincial environment is full of light, with an uneven seasonal distribution of precipitation [16]. The annual average precipitation is 550–950 millimeters with a decrease from the southeast to northwest. It rains mainly during the summer.

### Hand, foot and mouth disease data and meteorological data

The incidence of HFMD is seasonal. May through July and September through October experience the annual peak incidences of HFMD [17]. The incubation period of HFMD is 3–7 days [18]. Therefore, the statistics presented here for the HFMD incidence in each county of Shandong Province in 2008 during the aforementioned peak incidence months were for 21 weeks. CDC China granted us permission to use the HFMD incidence data. We used meteorological data from 756 national meteorological stations in 2008 from April through October, obtained from the China Meteorological Data Sharing Service System (http://cdc.cma.gov.cn). Kriging spatial interpolation in the ArcGIS Spatial Analyst tools module was used to generate meteorological data for national counties, according to the longitude and latitude of each meteorological station. For the weekly scale, the overall distribution of the meteorological data and incidence rates in all of the counties are shown in Table 1. National meteorological data (average wind speed, precipitation, average temperature, average temperature, RH) were sorted according to buffer radii of 10, 20, 30, 40, 50, 60, 70, 80, 90 and 100 kilometers within the counties, for the prior 1 to 8 weeks. Prior to SVD analysis, all of the data were processed into a rectangular flat form.

**Table 1:** **Descriptive statistics of the incidence rate and meteorological data**

	**Min**	**Max**	**Mean**	**Standard deviation**
incidence rate (1/100000)	0	558	8.3	25.26
average wind speed (0.1 m/s)	10.94	54.97	24.17	5.99
precipitation (0.1 mm)	0	251.99	34.57	38.86
average temperature (0.1°C)	71.81	276.04	211.07	39.24
temperature difference (0.1°C)	42.66	138.59	89.62	19.82
RH (1%)	39.89	92.76	72.49	10.14

### Research concept

First, we performed SVD analysis with meteorological elements at different spatiotemporal scales and HFMD incidence rates in the counties of Shandong. Then, we used the randomized trials method proposed by Wallace [19] to remove meteorological elements having spurious correlations with the incidence. In this manner, we obtained elements actually associated with the incidence. Finally, to find a region of significant correlation, we adopted the SVD method for meteorological elements and HFMD, the first modal time correlation coefficients of which were maximal. To explain the rationale for correlation with the incidence rate of meteorological factors during the prior two weeks, we performed SVD analysis between wind speed during the prior two weeks in counties within the 50-km buffer circle of Shandong Province and wind speed during the prior one week in these counties.

### SVD method

The SVD method was originally developed to diagnose structures between two meteorological fields [20-23]. Jiang and Ding theoretically proved that SVD was generally applicable to diagnostic analysis of the temporal and spatial distribution of coupled meteorological fields [21]. SVD is based on the maximum covariance of the two fields, and it calculates singular values of cross-covariance matrices, left singular vectors, right singular vectors, and time coefficients. Pairs of singular vectors and corresponding expansion coefficients constitute pairs of SVD spatial modes. Because of its solid mathematical foundation and its simple principles and calculations and the easily comprehensible physical meaning of the coupling signal, SVD is particularly suitable for teleconnection studies in meteorological fields. In recent years, SVD has been widely used in climate diagnostic studies, which have obtained valuable results [21,23].

In practical application, there are two data fields that respectively include spatial points p and q. There are observed values after a moment of f rectangular flat or standardized treatment by n times, expressed as a matrix:

1 $$ X=\left(\begin{array}{cccc}\hfill {x}_{11}\hfill & \hfill {x}_{12}\hfill & \hfill \dots \hfill & \hfill {x}_{1n}\hfill \\ {}\hfill {x}_{21}\hfill & \hfill {x}_{22}\hfill & \hfill \dots \hfill & \hfill {x}_{2n}\hfill \\ {}\hfill \vdots \hfill & \hfill \vdots \hfill & \hfill \dots \hfill & \hfill \vdots \hfill \\ {}\hfill {x}_{p1}\hfill & \hfill {x}_{p2}\hfill & \hfill \dots \hfill & \hfill {x}_{pn}\hfill \end{array}\right)\kern1.8em Y=\left(\begin{array}{cccc}\hfill {y}_{11}\hfill & \hfill {y}_{12}\hfill & \hfill \dots \hfill & \hfill {y}_{1n}\hfill \\ {}\hfill {y}_{21}\hfill & \hfill {y}_{22}\hfill & \hfill \dots \hfill & \hfill {y}_{2n}\hfill \\ {}\hfill \vdots \hfill & \hfill \vdots \hfill & \hfill \dots \hfill & \hfill \vdots \hfill \\ {}\hfill {y}_{q1}\hfill & \hfill {y}_{q2}\hfill & \hfill \dots \hfill & \hfill {y}_{qn}\hfill \end{array}\right) $$

X and Y are the left and right fields. A cross covariance matrix of the two fields is

2 $$ {C}_{XY}=\left\langle X{Y}^T\right\rangle $$

The symbol 〈〉 indicates averaging. The left and right fields are transformed using two orthogonal linear transformations, so the covariance is maximized. The two linear transformation matrices are *L*^*T*^ and *R*^*T*^; in other words, under conditions *LL*^*T*^ = *I* and *RR*^*T*^ = *I* (I is the unit matrix),

3 $$ Cov\;\left({L}^T\;X,\;{R}^T\;Y\right)={L}^T{C}_{XY}R= Max $$

This satisfies the conditions of L and R, according to linear algebra theory, and yields

4 $$ {C}_{XY}=L\left|\begin{array}{l}{\displaystyle \sum}\\ {}0\end{array}\right.\left.\begin{array}{l}0\\ {}0\end{array}\right|{R}^T $$

∑ = *diag*(*δ*_1_, *δ*_2_,......, *δ*_*m*_) is the diagonal, *δ*_1_ ≥ *δ*_2_≥...... ≥ *δ*_*m*_ > 0,which is the square root of the nonzero eigenvalue of ${C}_{XY}{C}_{XY}^T$, *m* ≤ min(*p*, *q*), and Equation (4) is the singular value decomposition of the matrix. *L* is the left singular vector of *C*_*FG*_, and R is its right singular vector. The L and R in column k(*l*_*k*_ and *r*_*k*_) ( *k* = 1,2,......,*m*) are called the k pairs of space.

Suppose *A* = *L*^*T*^*X* and *B* = *R*^*T*^*Y*_:_

5 $$ A=\left(\begin{array}{l}{a}_1\\ {}{a}_2\\ {}.\\ {}.\\ {}.\\ {}{a}_p\end{array}\right)\kern1.08em B=\left(\begin{array}{l}{b}_1\\ {}{b}_2\\ {}.\\ {}.\\ {}.\\ {}{b}_q\end{array}\right) $$

A and B are time-coefficient matrices of left field X and right field Y. *a*_*k*_ and *b*_*k*_ are k-mode time coefficients of X and Y, respectively. Each space and corresponding time coefficient determines a modal. After obtaining the time coefficient matrix, we can define the correlation coefficient between every pair of singular vectors of the time coefficients *r*_*k*_(*A*, *B*) (k is the number of the corresponding modal). *r*_*k*_(*A*, *B*) is the degree of relationship of two fields in every modal, reflecting the overall status of the typical variable field [24]:

6 $$ SC{F}_k={\sigma}_k^2/{\displaystyle \sum_{i=1}^m{\sigma}_i^2} $$

This equation yields the K modal covariance characterization interpretation of the total covariance proportion. The K mode is more important with greater *SCF*_*k*_:

7 $$ CSC{F}_N=\frac{{\displaystyle \sum_{i=1}^N{\sigma}_i^2}}{{\displaystyle \sum_{i=1}^m{\sigma}_i^2}} $$

This equation yields the N modal covariance characterization interpretation of the total covariance proportion (N ≤ m). Because *δ*_1_ ≥ *δ*_2_≥...... *δ*_*m*_ > 0, *CSCF*_*N*_ is larger, indicating that the N mode of the total covariance is larger, and the relationship between the expansion coefficients can represent that which lies between the left and right fields.

Because the covariance between A and B is maximized, the order of the expansion coefficients is arranged by pairs of covariance sizes. The large covariance generally focuses on the front N pairs, and the remaining covariances are small. We can choose the first N expansion coefficients for which the first N relationships between the expansion coefficients can substantially represent the relationship between the two fields. Thus, these coefficients can reduce the variable dimension, find the major large-scale coupled signals of left-field and right-field interaction, and filter out small-scale random perturbations. This process is the principle of SVD analysis in exploring the relationship between two main fields [25].

To explore the relationships between meteorological elements on different spatiotemporal scales and HFMD incidence in Shandong Province, the latter variable is used as the right field, and the spatial and temporal scale of meteorological elements is used as the left field. The paired SVD mode with HFMD in Shandong and meteorological elements at varying spatiotemporal scales can be contained with the SVD method. In this manner, we can analyze the strongest characteristics of spatiotemporal variation. We used the randomized trial method proposed by Wallace to test significance of the SVD mode [19] — two fields use the sample data; one is based on the actual number of data in chronological order, and the other is in random order. That is, samples of the two fields do not match in time. In this case, we obtained the total squared covariance of the two fields:

8 $$ {\left\Vert {C}_{xy}\right\Vert}^2={\displaystyle \sum_{k=1}^m{\sigma}_k^2} $$

The k pairs of the SVD mode to the total sum of squares covariance contribution rate is

9 $$ {S}_k=\frac{\sigma_k^2}{{\left\Vert {C}_{xy}\right\Vert}^2} $$

To obtain ‖*C*_*xy*_‖^2^ and *S*_*k*_ in a series of randomized trials, we repeat replacement of a sample number in the second field randomly, for a total of 100 times. Then, we compare ‖*C*_*xy*_‖^2^ and *S*_*k*_, which are obtained using data corresponding to the actual time with a series of randomized trials. If ‖*C*_*xy*_‖^2^ obtained with the actual data is greater than ‖*C*_*xy*_‖^2^ obtained with SVD 100 randomized trials in the position 5 maximum, one can assume a relationship between the two variable fields. In this context, for the *k*-th mode, if *S*_*k*_ obtained with the actual data is greater than *S*_*k*_ obtained with SVD 100 randomized trials in the position 5 maximum, then the *k*-th mode indicates that the relevant link is significant [25].

## Results

Upon SVD analysis with meteorological elements on different spatiotemporal scales and HFMD incidence in Shandong Province counties, only wind speed and RH satisfied the α = 0.05 significance level of the Monte Carlo test. Therefore, we only analyzed the relationships between wind speed and RH and HFMD incidence.

**Figure 1:**
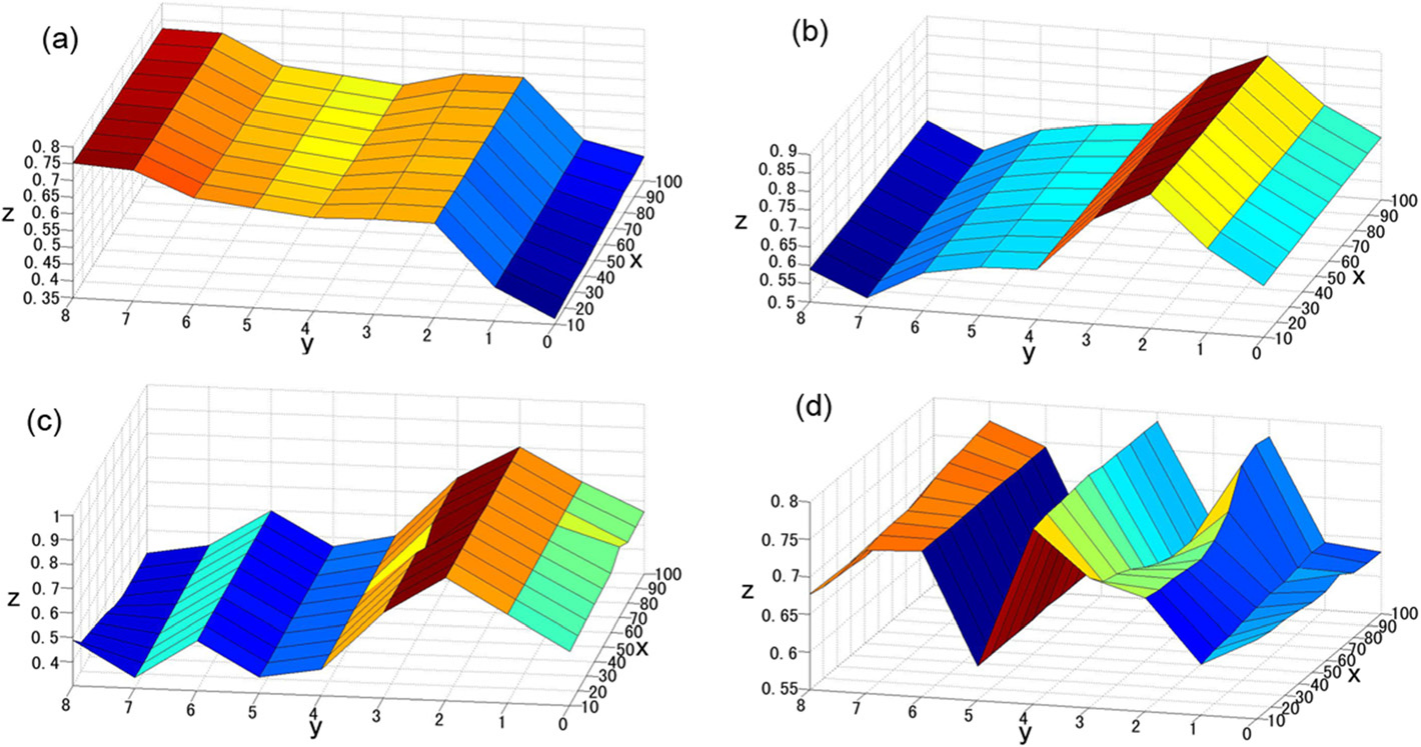
**First modal time correlation coefficient of SVD between meteorological elements and HFMD incidence. (a)** RH. **(b)** wind speed. **(c)** wind speed from May through July. **(d)** wind speed from September through October. x: buffer radius (km), y: number of weeks in advance, z: correlation coefficient.

Figure 1 shows the first pair of modal-state time correlation coefficients. These coefficients were obtained from SVD analysis using wind speed and RH within different circular buffers and different periods and HFMD incidence in the province (geometric centers of circles were used to create buffers within the counties of Shandong). The x-axis is the buffer radius (in kilometers), the y-axis is the number of weeks prior, and the z-axis is the first pair of modal-state time correlation coefficients.

Figure 1(a) shows time correlation coefficients between RH on various spatiotemporal scales and HFMD morbidity in Shandong Province. The figure shows that when the number of weeks prior was the same, the correlation coefficients in buffers of different radii did not fluctuate much. Within the same buffer radius, the correlation coefficient increased with a few additional prior weeks. Two and seven weeks prior were the inflection points, consistent with the findings of Wang [26]. The correlation coefficient increased for less than two weeks prior. The SVD first modal time correlation coefficient between RH within the 10-km buffer circle of the counties of Shandong with an eight-week lag and the HFMD incidence in Shandong was the maximum.

Figure 1(b) shows the time correlation coefficient between wind speed on different spatiotemporal scales and HFMD morbidity in Shandong Province. The figure shows that when the number of weeks prior was the same, the correlation coefficients in buffers of different radii did not vary much. Within the same buffer radius, the correlation coefficients showed large fluctuations with more than a few weeks prior. Two weeks prior was an inflection point, as the correlation coefficient for less than this period increased greatly. The SVD first modal time correlation coefficient between wind speed within the 50-km buffer circle of the counties of Shandong with a two-week lag and the HFMD incidence in Shandong was the maximum.

**Figure 2:**
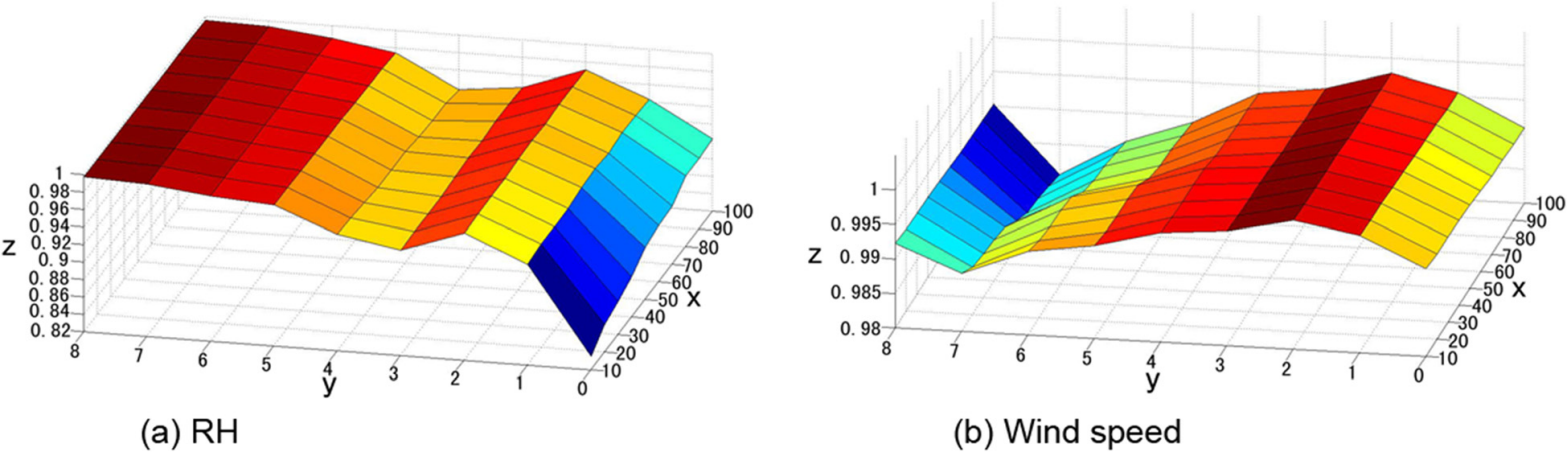
**First modal contribution rate of SVD between meteorological elements and HFMD incidence.** x: buffer radius (km), y: number of weeks in advance, z: first modal contirbution rate.

Figure 1(c) presents the time correlation coefficient between wind speed from May through July at different spatiotemporal scales and HFMD morbidity in Shandong Province, similar to Figure 2(b). The figure shows that when the number of weeks prior was the same, the correlation coefficients in buffers of different radii did not vary much. Within the same buffer radius, the correlation coefficients had fluctuations for more than a few weeks prior. Two weeks prior was a turning point, as correlation coefficients increased for periods shorter than that period.

Figure 1(d) shows the time correlation coefficients between wind speed from September through October on different spatiotemporal scales and HFMD morbidity in the province. The figure shows that when the number of weeks prior was the same, the correlation coefficients in buffers of different radii did not fluctuate widely. Within the same buffer radius, the correlation coefficients had fluctuations for more than a few weeks prior. Two and four weeks prior were the turning points.

The meteorological values in certain areas were continuous and stable, so the z-axis was insensitive to distance with the same number of weeks prior. Time correlation coefficients between wind speed on different spatiotemporal scales and HFMD morbidity in the province from May through July (Figure 2(c)) and from September through October (Figure 2(d)) were similar to the coefficients between those two variables in Figure 2(b), perhaps because wind speed and the incidence from September to October changed little. As a result, the wind speed could not be divided into two periods.

Figure 2 shows the first modal contribution rate obtained from SVD analysis between meteorological elements within various circular buffers and during different periods and HFMD incidence in the province. Only wind speed and RH satisfied the α = 0.05 significance level of the Monte Carlo test. The variance contribution rate of the first singular vectors was greater than 50%, much higher than the second and third modal variance contribution rates. In other words, the first space could explain the main information between the right and left fields. Figure 1 shows that the SVD first modal time correlation coefficient between wind speed within the 50-km buffer circle of the Shandong counties with two-week lag and the HFMD incidence in Shandong was the maximum. The SVD first modal time correlation coefficient between RH within the 10-km buffer circle of the counties with eight-week lag and the incidence was the maximum. Therefore, we performed SVD analysis between wind speed within the 50-km buffer circle of the counties with two-week lag (left field) and the HFMD incidence in Shandong (right field). We also performed SVD analysis between RH within the 10-km buffer circle of the counties with eight-week lag (left field) and the HFMD incidence (right field). To explain the rationality of correlating meteorological factors within two weeks prior with the incidence, we performed SVD analysis of wind speed for two weeks prior in the counties within the 50-km buffer of Shandong Province and wind speed for one week prior in these counties.

**Figure 3:**
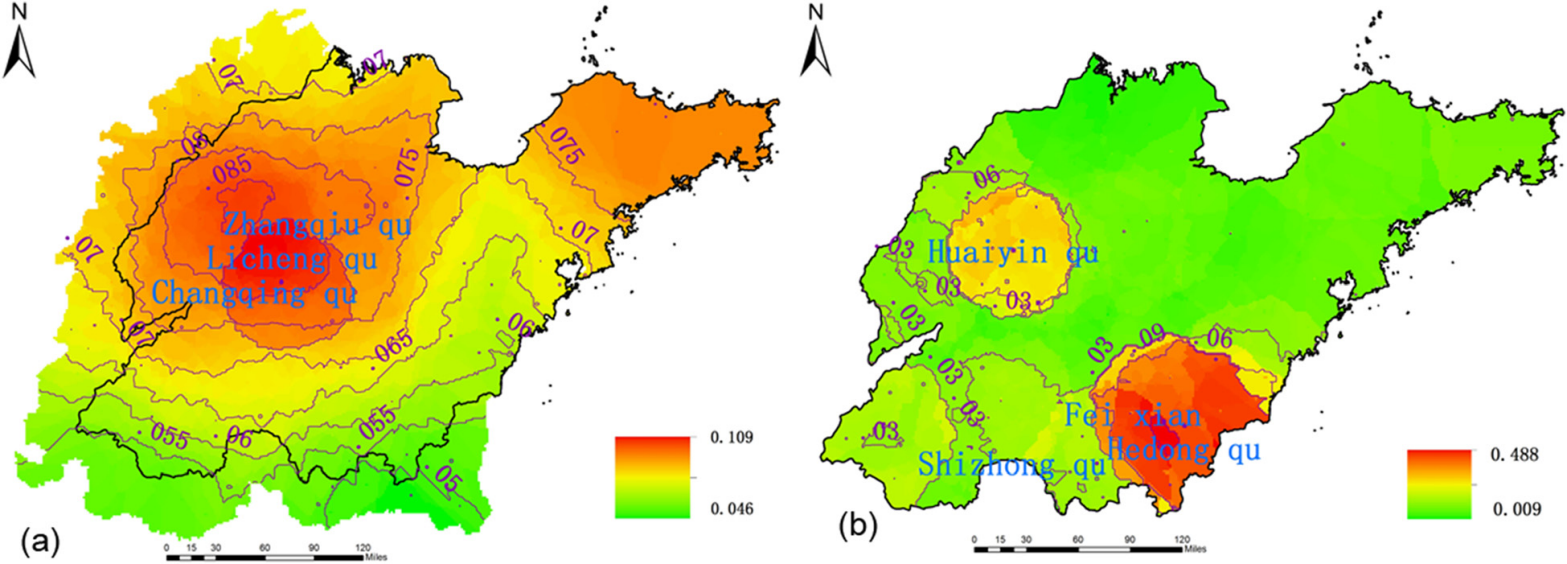
**First modal singular vectors of SVD analysis between wind speed and HFMD incidence. (a)** First modal left singular vectors (wind speed) of SVD analysis. **(b)** First modal right singular vectors (prevalence) of SVD analysis.

Based on SVD analysis between wind speed within the 50-km buffer circle of Shandong counties prior to two weeks and the HFMD incidence in Shandong, Figures 3(a) and 3(b) show the first pair of SVD analysis modes between wind speed within the 50-km buffer circle of Shandong counties for two weeks prior (left field) and HFMD incidence, which satisfied the α = 0.05 significance level of the Monte Carlo test and which had a modal time correlation coefficient of 0.864. Thus, the variance of the first pair of singular vectors was large, which could represent the coupling relationship between wind speed and HFMD incidence. The corresponding wind speed mode is shown in Figure 3(a) in which there was a positive anomaly with high-value centers in northwestern Shandong Province. The disease field in the province (Figure 3(b)) showed positive anomalies, particularly in the northwest and southeast areas, with maximum values showing that when wind speed in the northwest during two weeks prior was anomalously small (large), the HFMD incidence there and in the southeast was abnormally small (large).

**Figure 4:**
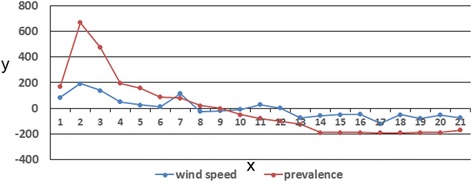
**First modal time coefficient of SVD analysis between wind speed and HFMD incidence in Shandong Province.** x: number of weeks in advance, y: time coefficient.

The above analysis indicated that wind speed within the 50-km buffer circle of Shandong counties for two weeks prior and HFMD incidence in Shandong had consistent positive anomalies. This finding clearly illustrated that the wind field and disease field anomalies underwent similar changes in the region. Figure 4 shows the time coefficients of the first left and right singular vectors. Evolution of these two vectors showed high consistency, with a correlation coefficient reaching 0.864, which satisfied the α = 0.05 significance level of the Monte Carlo test. Figure 4 shows that time coefficient changes in the first pair of singular vectors were consistent, but their amplitudes were not identical. The change in the wind speed field amplitude was less than that in the disease field.

(1) SVD analysis of wind speed for two weeks prior in counties within the 50-km buffer circle of Shandong Province and wind speed for one week prior in Shandong counties.

**Figure 5:**
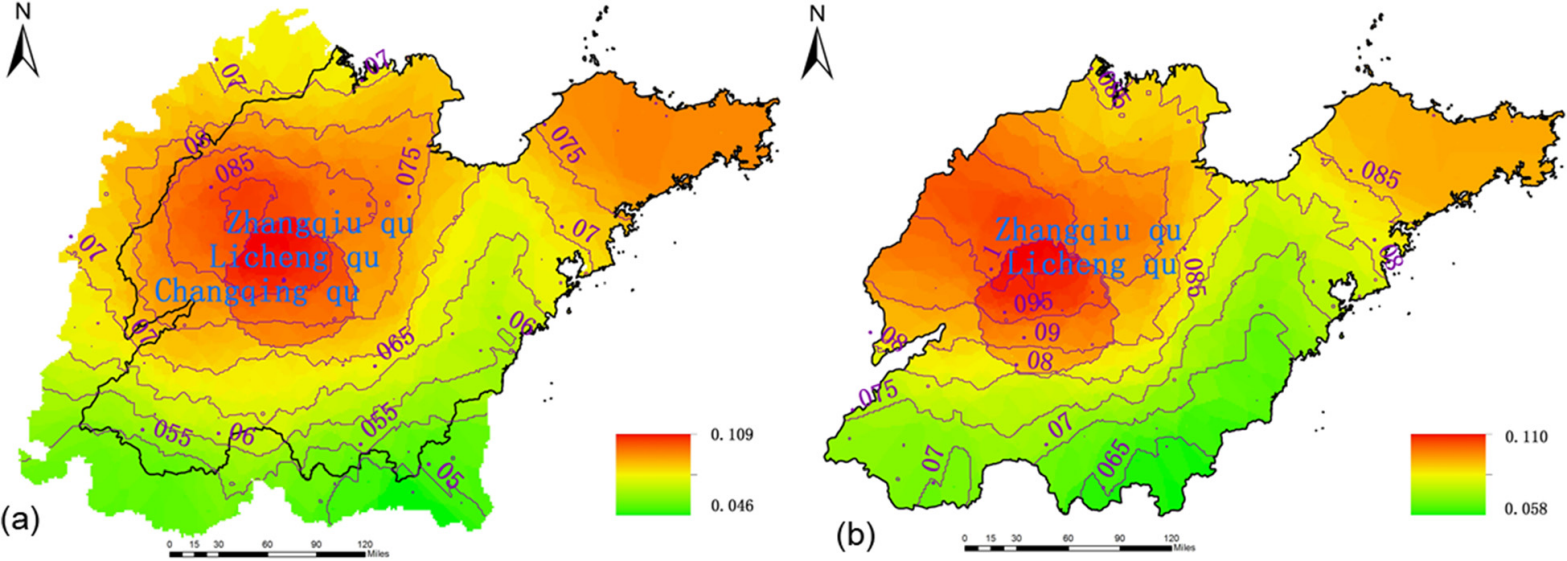
**First modal singular vectors of SVD models between wind speed for two weeks prior in the counties within the 50-km buffer circle of Shandong Province and wind speed for one week prior in the counties. (a)** First modal left singular vectors of SVD analysis. **(b)** First modal right singular vectors of SVD analysis.

Figures 5(a) and 5(b) display the first pair of SVD analysis modes between wind speed for two weeks prior in the counties within the 50-km buffer circle of Shandong Province and wind speed for one week prior in the these counties. The pair satisfied the α = 0.05 significance level of the Monte Carlo test, their variance accounted for 99% of the total variance, and the modal time correlation coefficient was 0.67. Thus, the variance of the first pair of singular vectors was large, indicative of the coupling relationship between wind speed for two weeks prior in the counties within the 50-km buffer circle of the province and wind speed for one week prior in these counties.

Figures 5(a) and 5(b) show a significant positive correlation between wind speed for two weeks prior in the counties within the of 50-km buffer circle of the province (left field) and wind speed for one week prior in these counties (right field). The related regional pattern was consistent in the northwestern part of the province. That is, when wind speed for two weeks prior in the counties within the 50-km buffer circle in the northwest province was anomalously small (large), wind speed for one week prior in the Shandong counties was abnormally small (large).

**Figure 6:**
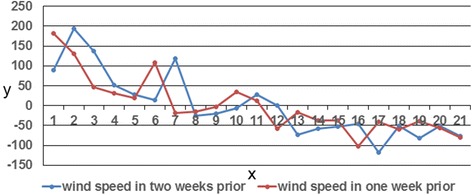
**First modal time coefficient of SVD analysis between wind speed for two weeks prior in the counties within the 50-km buffer circle of Shandong Province and wind speed for one week prior in the counties.** x: number of weeks in advance, y: time coefficient.

The above analysis indicated that wind speed within the 50-km buffer circle of the Shandong counties for two weeks prior (left field) and wind speed in these counties for one week prior (right field) showed consistent positive anomalies. This finding clearly illustrated similar changes in the anomalies in the wind field for two weeks prior and for one week prior. Figure 6 shows the time coefficients of the first left and right singular vectors. Evolution of these two vectors showed high consistency, with a correlation coefficient reaching 0.67 and satisfaction of the α = 0.05 significance level of the Monte Carlo test. Figure 6 shows that the change in time coefficient of the first pair of singular vectors was consistent.

(2) SVD analysis between RH within the 10-km buffer circle of Shandong counties for eight weeks prior and HFMD incidence in Shandong.

**Figure 7:**
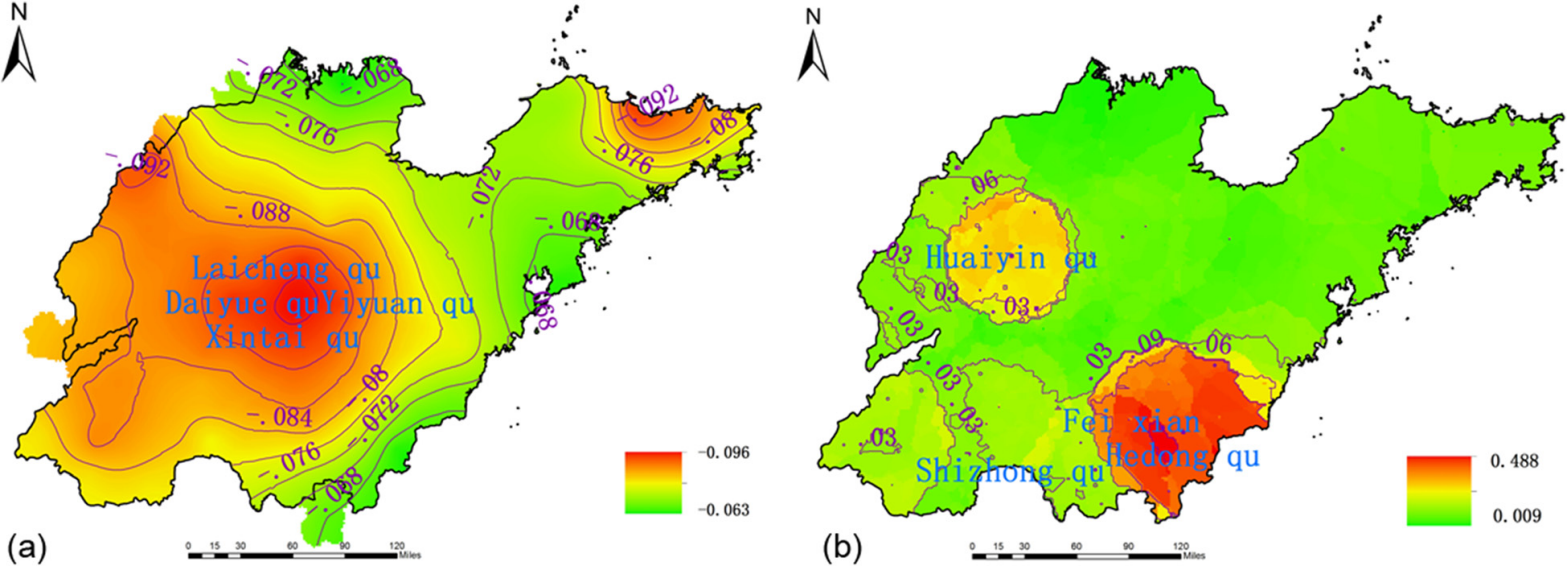
**First modal singular vectors of SVD analysis between RH and HFMD incidence. (a)** First modal left singular vectors (RH) of SVD analysis. **(b)** First modal right singular vectors (prevalence) of SVD analysis.

Figures 7(a) and 7(b) present the first pair of SVD analysis modes between RH within the 10-km buffer circle of the province counties for eight weeks prior (left field) and the HFMD incidence in Shandong (right field). This pair satisfies the α = 0.05 significance level of the Monte Carlo test, and their variance accounted for 99.7% of the total; the modal time correlation coefficient was 0.7497. Thus, the variance of the first pair of singular vectors was sufficiently large to indicate a coupling relationship between RH and HFMD incidence. The corresponding RH mode is shown in Figure 7(a), with a negative anomaly with high-value centers in the central province. The province disease field (Figure 7(b)) showed positive anomalies, particularly in the northwest and southeast, indicating that when RH in the central region during the prior eight weeks was anomalously small (large), the HFMD incidence rates in the northwest and southeast province were abnormally large (small).

**Figure 8:**
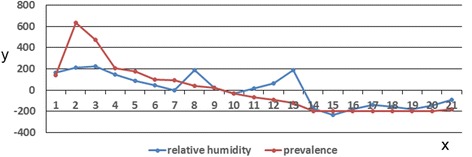
**First modal time coefficient of SVD analysis between RH and HFMD incidence in Shandong Province.**

The above analysis indicated that RH within the 10-km buffer circle of the Shandong counties for eight weeks prior (left field) had consistent negative anomalies, corresponding to consistent positive anomalies of HFMD incidence in Shandong. This finding clearly showed that wind field and disease field anomaly changes were opposite within the region. Figure 8 shows time coefficients of the first left and right singular vector. This figure reveals that the relationship between the two fields was strong and that the change in the time coefficients of the first pair of singular vectors was consistent.

## Discussion

Over the past decade, there have been frequent and widespread HFMD outbreaks in Asian countries. Therefore, researchers have applied many mathematical methods to analyze the relationships between meteorological factors and disease. Huang analyzed the associations between weekly HFMD cases and meteorological variables, using the generalized additive model and the time-series method [14]. However, spatial effect was considered in this study. Khormi used the Getis-Ord Gi* statistic to illustrate the impact of different spatiotemporal scales on dengue fever control decisions [15]. The Getis-Ord Gi* statistic is a traditional spatial clustering method. It is used to identify and visualize hotspots and geographic homogeneity, rather than to explore the driving factor [27]. Hu used geographically weighted regression models to explore the associations between meteorological factors and HFMD incidence [12]. Allowing for spatial variation in the relationships between variables across the study area, geographically weighted regression models could capture the average strength and significance of statistical relationships between factors and HFMD only at a certain time cross-section [28].

However, although the incubation period of HFMD only ranges from 1 to 7 days, the viruses that cause HFMD can exist in the bodies of asymptomatic carriers, infected persons, and persons who have only recently recovered. A long contagious period of HFMD could cause clinical presentations to be detected late [9]. Therefore, it is significant for disease control and prevention to model both the spatial and temporal lag effects of weather on HFMD risk. In this study, we introduced the SVD method to evaluate the effects of meteorological anomalies on different spatiotemporal scales on HFMD incidence. The SVD method could reduce a dataset with many values to a dataset with significantly fewer values but that retains most of the original data variability. Data compression in the SVD analysis resulted in a more compact representation of the correlations between meteorological factors and HFMD incidence, and it could provide insight into the spatial and temporal variations exhibited in the fields of data being analyzed [29].

We observed a positive relationship between average wind speed and HFMD incidence, consistent with findings of other recent studies conducted in Hong Kong [8]. Our results also indicated a negative association between average RH and HFMD incidence, which was not consistent with other findings [9]. A report from the Kaifeng City Observatory showed that the monthly average RH there during peak disease periods was between 50% and 62% [10], which is lower than the mean average RH in Shandong Province during the study period (Table 1). There might be a threshold effect of average RH on HFMD incidence. When monthly average humidity is >62% or <50%, HFMD might be inhibited [10]. The average RH in Shandong exceeded this threshold and might therefore have inhibited HFMD.

Meteorological factors are important effects of regional climate, and they impact the incidence and development of infectious diseases. These factors not only affect the body’s immune system, but they can also greatly influence biological pathogens in the environment and mediate biological reproduction and dissemination, thereby playing an important role in the incidence of infectious diseases in populations [30]. Wind speed is a risk factor for HFMD. Many respiratory viruses generally survive for a short period of time in the external environment. Wind speed can help to dilute airborne concentrations of some viruses to a certain extent, thereby reducing the danger of infection. However, HFMD viruses are different from these disease viruses in that they are strongly resistant and can survive for up to one year in the external environment. They are easily transported in dust blown by the wind. Wind can contribute pollutants and accelerate the propagation velocity of droplets, spreading the HFMD virus. The more developed the region, the more prevalent HFMD is, perhaps because economically developed areas have greater population densities and more mobile populations, thereby increasing opportunities for contact between individuals and promoting the spread of HFMD [8,31].

We found that HFMD incidence anomalies were better explained when wind speed anomalies were used with a two-week lag, which was not surprising and was consistent with previous findings in Hong Kong [8]. The incubation period of HFMD is 1–7 days [9]. There was a positive correlation between wind speed for two weeks prior in Shandong Province counties within the 50-km buffer circle and wind speed for one week prior in those counties. Furthermore, wind speed for one week prior affected HFMD morbidity. Additionally, potential delays in parents becoming aware of clinical symptoms of the disease in their children [15] and the time-consuming process of patients seeking hospital treatment [32] affect the disease with certain time lags. Wind speed for two weeks prior in the counties within the 50-km buffer circle of Shandong Province affected HFMD morbidity.

Our results showed that HFMD incidence anomalies were also better explained by RH anomalies within the period of eight weeks prior. Our results confirmed those of previous time-series analyses that suggested an association between HFMD and humidity in Japan, Hong Kong, and another city, Rizhao, of Shandong Province [9,33]. However, the effects and kinetic mechanisms of the RH anomaly during the prior eight weeks on HFMD morbidity abnormality are unknown.

## Conclusions

It is of great importance to detect anomalies in HFMD incidence in a timely manner. This study provided evidence of strong associations of HFMD incidence in children with wind and RH. Thus, meteorological anomalies two or eight weeks prior could be used as valid references to detect abnormalities during infectious disease peak periods. The study result could furnish a valid reference for policy makers and health agencies in detecting abnormalities in disease peak periods, thereby improving surveillance capabilities and the selection of appropriate prevention and control strategies. This study was the first in China using the SVD method to evaluate the effects of meteorological anomalies on different spatiotemporal scales on HFMD incidence. SVD could be applied not only to HFMD but also to other infectious diseases. A limitation of the present study was the buffer radius used because the scope of influence of meteorological elements lacked certain criteria, which could have affected the accuracy of the results. In addition, to understand the effects and kinetic mechanism of wind and RH anomalies on HFMD morbidity exceptions, further analysis and research are needed.
